# Surface Mechanical Characterization of Carbon Nanofiber Reinforced Low-Density Polyethylene by Nanoindentation and Comparison with Bulk Properties

**DOI:** 10.3390/nano9101357

**Published:** 2019-09-22

**Authors:** Peyman Nikaeen, Dilip Depan, Ahmed Khattab

**Affiliations:** 1 Mechanical Engineering Department, College of Engineering, University of Louisiana at Lafayette, P.O. Box 43675, Lafayette, LA 70504-4130, USA; peyman.nikaeen@gmail.com; 2 Chemical Engineering Department, College of Engineering, University of Louisiana at Lafayette, P.O. Box 43675, Lafayette, LA 70504-4130, USA; 3 Industrial Technology Department, College of Engineering, University of Louisiana at Lafayette, P.O. Box 43675, Lafayette, LA 70504-4130, USA; khattab@louisiana.edu

**Keywords:** LDPE, nanoindentation, carbon nanofiber, surface mechanical characterization, Halpin-Tsai model

## Abstract

Surface mechanical properties of low-density polyethylene (LDPE) reinforced by carbon nanofibers (CNFs) up to 3% weight load were investigated using nanoindentation (NI). Surface preparation of the nanocomposite was thoroughly investigated and atomic force microscopy (AFM) was used to analyze the surface roughness of the polished surfaces. The dispersion of nanofillers in the LDPE matrix was examined using scanning electron microscopy (SEM). The effect of various penetration loads on the results and scattering of the data points was discussed. It was found by NI results that the addition of 3% weight CNF increased the elastic modulus of LDPE by 59% and its hardness up to 12%. The nano/micro-scale results were compared with macro-scale results obtained by the conventional tensile test as well as the theoretical results calculated by the Halpin-Tsai (HT) model. It was found that the modulus calculated by nanoindentation was twice that obtained by the conventional tensile test which was shown to be in excellent agreement with the HT model. Experimental results indicated that the addition of CNF to LDPE reduced its wear resistance property by reducing the hardness to modulus ratio. SEM micrographs of the semicrystalline microstructure of the CNF/LDPE nanocomposite along with the calculated NI imprints volume were examined to elaborate on how increasing the penetration depth resulted in a reduction of the coefficient of variation of the NI data/more statistically reliable data.

## 1. Introduction

Nanoindentation is a powerful non-destructive testing technique to evaluate the mechanical properties of materials such as elastic modulus, hardness and creep. It offers the analysis of small size specimens such as micro and nano-size structures, thin films and coatings [1] where conventional testing techniques are impractical. Since introduction of nanoindentation, it has been extensively used to find mechanical properties of different systems such as MEMS devices [2,3], functionally graded materials, biomedical materials [4] and polymer nanocomposites [5,6]. It has been successfully used to mechanically characterize the in situ constituents and interphases in fiber reinforced polymer composites allowing direct comparison between different polymer matrix blends and fiber treatments [7,8,9,10].

Rapid advances in modern technologies demand the development of more robust and versatile functional materials and components. Polymer composites reinforced with carbon nano-fillers are increasingly finding attention due to their enhanced properties such as excellent strength and stiffness, high strength to weight ratio, high thermal stability, and superior electrical properties [11]. Low-density polyethylene (LDPE), a thermoplastic polymer with low crystallinity and long chain branching, has attracted the attention of researchers as an excellent candidate owing to its excellent bubble stability and high melting strength [12]. It has a broad range of applications such as in automotive and aerospace industry as thermal blankets, radiation shielding, circuit boards, and insulation films [13,14,15]. However, it has some drawbacks like low strength, elastic modulus and hardness, which can be improved by introducing carbon nanofillers [16]. Some researchers studied the effect of adding carbon nanofiller to a LDPE matrix. Majeed et al. [17] compared adding three different nanotubes (titania, halloysite & CNT) to LPDE and concluded that among the three, the addition of CNT showed the best improvement in tensile and thermal properties of LDPE. Khattab et al. [18] found that adding 3 wt. % CNFs enhanced the elastic modulus of LDPE by 44%. Both the aforementioned studies were focused on the macro-level mechanical properties of CNF/CNT-reinforced LDPE using conventional tensile testing methods. 

There are some researches on the nanoindentation of LDPE: Bouaita et al. [19] conducted NI on five different polyolefin including LDPE and measured the storage modulus of LDPE as 0.334 GPa. It was well discussed that due to the existence of morphological features with sizes smaller than 10 μm in polymers, indentation size has a great effect on the measured properties. Bischel et al. [20] used nanoindentation to calculate the relative elastic modulus of the crystalline features and to identify phases within superstructures of the LDPE/HDPE blends. Jee et al. [21] measured the mechanical properties of LDPE disks with a thickness of 1 mm by AFM nanoindentation using the Oliver & Pharr method and image analysis and found that the two methods yield almost the same results. However, to the best of the authors’ knowledge, nanoindentation investigation of the CNF/LDPE nanocomposite has not yet been reported. Moreover, none of the above-mentioned studies focused on the morphology of the LDPE nanocomposite and its effect on the NI results in details.

In this study, nanoindentation was employed to find the surface mechanical properties of the CNF-reinforced LDPE at the nano and micro-scale. Scanning electron microscopy was utilized to inspect the dispersion of fibers and morphology of the nanocomposite surface. Surface preparation of the composite, which is crucial to get acceptable and reliable indentation results, is thoroughly investigated, and atomic force microscopy (AFM) was used to analyze the surface roughness of the polished surfaces. Elastic modulus and hardness values are acquired at nano/micro level by nanoindentation for LDPE systems with different loadings of 0, 1, 2, and 3 wt. % of CNF. The effect of various penetration loads on the results and scattering of the data points is discussed. Finally, the nanoindentation results are compared with the macro-scale tensile test results from the literature as well as the theoretical ones obtained by the Halpin-Tsai model. 

## 2. Materials and Methods

### 2.1. Materials

The LDPE (EM-460AA) used in study is provided by Westlake Polymers Corporation (Houston, TX, USA). The mechanical properties of the LDPE were given by the provider with an ultimate tensile strength of 13.1 MPa, elastic modulus of 234.4 MPa, and the melt flow index of 37 g/10 min. Vapor-grown carbon nanofiber (VGCNF) were provided by Pyrograf Products, incorporation (Cedarviller, OH, USA). Its specific fiber identification is PR-24-XT-LHT. It has an average diameter of 100 nm and a length of 30 to 100 μm. The tensile modulus of this CNF is 600 GPa and has a tensile strength of about 7 GPa.

### 2.2. Fabrication of the Nanocomposite

CNF/LDPE nanohybrids were prepared using a previously-reported procedure [18]. Briefly, a rotary mixer was utilized at 60 rotations per minute (rpm) to blend the LDPE pellets with 0, 1, 2, and 3 wt. % of CNFs at room temperature for 40 min. Shear forces were applied by a single screw extrusion machine (Killion extruders) to disperse the CNFs into the LDPE. The rotating speed of the extruder was kept constant at 40 rpm with an operating temperature of 135 °C for the first barrel zone and 149 °C for the adaptor and die zones. After extrusion, water bath was used to cool down and solidify the composite. In the last step, the injection molding machine was used to prepare dog bone-shaped samples for tensile tests.

### 2.3. Scanning Electron Microscopy

The dispersion of CNFs in the LDPE matrix was investigated using JEOL 6300F field emission scanning electron microscope (SEM) located at the microscopy center of University of Louisiana at Lafayette, LA, USA. Samples were coated with 15 nm of gold and 15 kV and 5 kV beam voltages were used to capture micrographs at low and high magnifications, respectively.

### 2.4. Nanoindentation Studies

In this study, a nanoindenter G200 instrument provided by Agilent-MTS, which is compliant with ISO 14577, was used with a load range of 0.4–500 mN, displacement resolution of less than 0.01 nm, and a load resolution of 50 nN. The machine is equipped with a three-sided pyramidal-shaped Berkovich indenter with a tip radius of ~120 nm. In comparison to other tip geometries, Berkovich tips are much easier to manufacture due to the lack of a ‘chisel’ edge defect at the indenter tip and are less prone to wear [22]. Berkovich tips also induce plasticity at very small loads allowing hardness measurement. The importance of the treatment of the raw data and deriving properties have been studied by many researchers. The most commonly used method was developed by Oliver and Pharr [23]. According to their method, hardness is calculated as:

(1) $$H=\frac{P_{max}}{A_{c}}$$

where, $P_{max}$ is the maximum applied load, and $A_{c}$ is the projected contact area of the tip on the surface of the testing material. The contact area is calculated according to Oliver and Pharr method (OP method) by a polynomial function of contact depth, $h_{c}$:

(2) $$A_{c}=24.56h_{c}^{2}+C_{1}h_{c}^{1}+C_{2}h_{c}^{\frac{1}{2}}+C_{3}h_{c}^{\frac{1}{4}}+C_{4}h_{c}^{\frac{1}{8}}+\ldots +C_{8}h_{c}^{\frac{1}{128}}$$

Wear causes tip rounding and deviation from its ideal geometry. To compensate the tip rounding effect on the calculation of the contact area, C values were empirically calculated by an area function calibration operation. The tip area calibration was performed prior to the actual tests by means of 25 indentations on a reference material, fused silica (with elastic modulus of *E* = 72 GPa). The elastic modulus of the specimen was calculated from the reduced modulus, $E_{r}$, as:

(3) $$E=\left(1-\nu_{s}^{2}\right){\left[\frac{1}{E_{r}}-\frac{1-\nu_{i}^{2}}{E_{i}}\right]}^{-1}$$

where $\nu_{s}$ and $\nu_{i}$ are the Poisson’s ratio of the specimen and indenter, respectively. For a diamond tip, $\nu_{i}$ and $E_{i}$ are 0.07 and 1141 MPa, respectively, and $\nu_{s}$ was chosen as 0.35 for LDPE. To achieve statistically-reliable results, 25 indents were made on each sample and mean and standard deviations were reported. It has been indicated by numerous studies that time-dependent behavior of polymers may affect the measurement of elastic modulus and hardness using a nanoindentation technique [24,25,26,27,28]. Poor non-linear curve fitting of the unloading segment as well as overestimation of the elastic modulus have been attributed to the noticeable viscoelastic the polymers. The most apparent effect of time-dependent behavior occurs as a negative slope in the commencement of unloading section in the load-displacement curve which is often described as “nose” [29,30]. Adding a constant load segment between loading and unloading segments as well as high unloading rates has been proposed to minimize the time-dependent effects while indenting viscous materials and to obtain reasonable measurements [25,31]. Therefore, a 50 s holding time between loading and unloading steps was selected to prevent noticeable errors due to materials creep behavior. Moreover, a relatively high unloading rate of around 13.5 mN s^−1^ was programmed accordingly. A representative load-displacement graph of CNF/LDPE systems indented at 200 mN load is given in Figure S1 in the Supplementary Material section. All in all, to avoid the influence of any possible time-dependent behavior of the nanocomposites on the NI results, the time-dependent test settings were kept constant for all shallow and deep indentations to allow a meaningful comparative analysis.

During pile-up, the contact depth is greater than maximum indentation depth which leads to overestimated indentation modulus in NI measurements [24]. An SEM image of an indent is presented as Figure S2 in the Supplementary Material section, showing no evidence of pile-up, which indicates that no significant pile-up effect on the measured properties obtained by NI technique occurred. 

The existing residual stress fields have shown to affect the mechanical properties of composite materials measured by nanoindentation techniques [32,33,34,35]. Such stress fields are readily generated by inhomogeneous heat treatment, local plastic deformation or thermal expansion coefficient mismatch between the matrix and the fillers in composite materials. Therefore, it is of the utmost importance to consider residual stresses while performing NI. In this study, since the volume percentage of CNF is very small, the effect of thermal coefficient mismatch between LDPE and CNF is negligible. Moreover, it was previously found that mechanical polishing has no effect on the measured NI values for polymer composites [36,37]. Therefore, having the knowledge that all the CNF/LDPE samples in this study underwent the same thermal processing conditions and have no plastic deformation, and more importantly, all the indentations were performed on a plane surface parallel to the longitudinal central plane of the dog bone samples (i.e., a plane on which thermal history is virtually identical). It can be concluded that residual stresses have no effect in the NI investigation of the proposed CNF/LDPE systems. However, a more detailed quantitative analysis of residual stresses by nanoindentation in such heterogeneous polymer nanocomposite systems can be a great topic for future studies.

### 2.5. Surface Preparation

Surface preparation of the specimens is a critical step to get reliable and acceptable results by nanoindentation. As mentioned before, the indentation depth was used to measure the area of contact between the indenter and the specimen’s surface, and hence, directly affects the material properties by OP method. Since the natural roughness of the surface causes errors in determination of the contact depth, nanoindentation data acquired at shallow depths in scales of 10s of nanometers require a very fine surface roughness to ensure the repeatability, reliability, and correctness of the material properties. Generally, surface roughness should not be larger than 5% of the indentation depth at which the results are acquired. Therefore, for an indentation of 1000 nm depth, a specimen with a surface roughness of about 50 nm is required.

Polishing is a common practice to get acceptable surface finish results. To polish the samples, they were embedded in a 3:1 epoxy-hardener system in an 18-mm diameter cylindrical cup. ASTM standard E3-11 [38], which deals with preparation of the metallographic specimens, was used as a guide to find an optimal polishing procedure. In contrast to metals, polymers have very soft surfaces, and even small vibrations and defects during polishing would result in high variations in the resulting surface roughness. Difficulties arise when very hard particles of carbon nanofibers are present in the very soft matrix of the polymer, making it even harder to obtain an evenly polished surface without scratches. Moreover, the CNFs that are detached from the surface, if not taken out by lubricant, act in favor of generating more scratches. It was shown by Khanna et al. [9] that the large difference between material removal rates of fiber and the polymer matrix constituents lead to an uneven level of CNF and polymer relative to the surface, following surface preparation.

**Figure 1:**
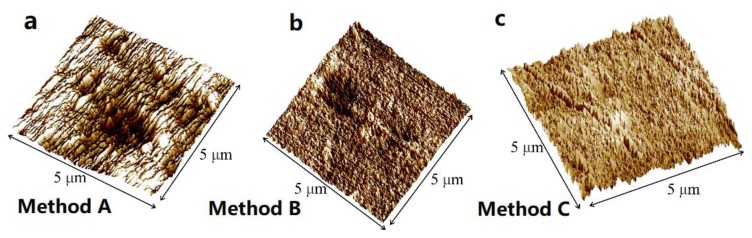
Sample of AFM scans of NI samples polished by (**a**) method A, (**b**) method B, and (**c**) method C. The details of the methods are given in Table 2.

In the current study, different grades of SiC papers were used successively to grind the surface prior to polishing. A manual polishing machine (Buehler ecomet-30) was utilized for grinding/polishing purposes, using water as a lubricant. The resulting surface finish was examined with AFM (Nanoscope IIIa by Digital Instruments) and quantitative analysis of the surfaces are given in Table 1 and Figure 1. In method A, only SiC papers were used to polish the samples and a mean surface roughness of about 50 nm (Figure 1a) was obtained which is not small enough for indentations below 1000 nm depth. Changing the water to an oil-based lubricant and repeating the steps with different rotating speeds and working times did not help to get a mirror-like surface finish using SiC papers. In method B, diamond suspension and alumina suspension with micro-cloths were used, resulting in relatively better surface finishes. The specimens were rinsed between each polishing/grinding step to remove any contaminants. All the suspensions were selected with neutral pH values to prevent possible reactions during the process.

The mean surface roughness achieved by method B was 28 nm, which in comparison to method A, showed significant improvement (Figure 1b). Due to the very soft characteristics of LDPE, scratches caused by polishing particles and residuals from the surface are almost inevitable and make it very hard to achieve mirror-like polishing results throughout the whole surface. However, practices such as decreasing the coarseness of the polishing steps gradually and avoiding aggressive polishing with high forces could benefit the results. Khanna et al. [39] found that increasing the polishing time to 40 min for monolithic polyesters gives better surface roughness. Therefore, in method C, alumina suspension with 0.3 micrometer particle size was used between the final steps (of the method B) and polishing times were increased. Consequently, better polishing results were observed (Figure 1c). The mean surface roughness by method C was 15 nm, which is acceptable for shallow indentations of about 300 nm depth. Table 2 presents all three methods with the steps and parameters of each in detail. Prior to nanoindentation and SEM analysis, polished samples were cleaned up in an ultrasonic cleaner to get rid of any polishing residues off the surface.

## 3. Results and Discussion

### 3.1. Surface Morphology

**Figure 2:**
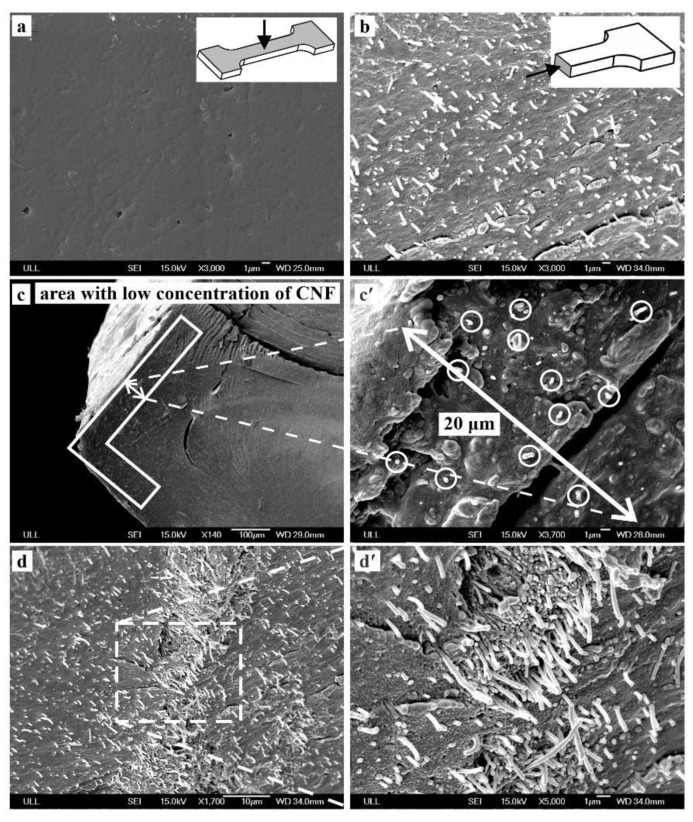
SEM micrographs of the (**a**) surface of the polished sample that shows no traces of CNF, (**b**) dispersion of CNFs on brittle fractured cross section, (**c**) very first microns of the surface from cross-sectional view which shows very few numbers of CNFs, (**d**) high agglomeration of CNFs in core part of the brittle fractured sample. All images are taken from a 3 wt. % CNF/LDPE sample and images (**c′**) and (**d′**) are magnified versions of a section of images c and d, respectively.

SEM micrographs for polished samples show no trace of CNFs, indicating that all CNFs are covered by LDPE during the grinding/polishing process (Figure 2a). Therefore, to investigate the dispersion of CNFs in LDPE in the injection molded samples, the cross-sectional area of the sample was used. A small piece of the sample was cut and kept in liquid nitrogen for 20 min to reduce its temperature, contributing to a brittle fracture along the cross-sectional area (Figure 2b–d). The micrographs indicated that the CNFs were more concentrated in the center of the sample (Figure 2d) and less prevalent in the extremities (Figure 2c). Figure 2c depicts that in the first 20 micrometers from the surface, the CNFs can barely be found, but while moving toward the center of the cross-sectional area, more fiber agglomerations were observed. 

The above observations indicated that during injection molding, CNFs tend to move toward the center of the cross-section rather than surface. This results in different modulus (E) and hardness (H) values between the surface and the core areas [40,41,42]. Moreover, different crystallinity exists between the outer and inner region of the sample as well. Crystallinity changes are associated with the temperature gradient effect induced by injection molding and also the uneven distribution of carbon nanofillers between the two regions [41]. In summary, SEM observations indicated that the dispersion of the CNFs in the LDPE matrix is inconsistent and inhomogeneous. Therefore, nanoindentation (NI) samples were prepared in a way to make sure that at least 20 micrometers of the surface were removed during grinding and polishing.

### 3.2. Nano-Mechanical Properties

Surface mechanical properties of the CNF-LDPE nanocomposite were studied using the grid indentation approach on a grid of 5 × 5 points. The grid indentation approach while using a correct depth and large number of experiments provides valuable information about the composite microstructure, morphological arrangement, and volumetric proportions of each mechanically dissimilar phase [43]. 

Since mechanical properties of a polymer obtained by NI are sensitive to penetration depth and to avoid indentation size effect at shallow depth, a basic force control method was employed to measure the mechanical properties of the nanocomposite at a certain resulting depth. This allows for an appropriate statistical analysis and comparison of the data between samples at a certain load. Due to Pharr et al. [44], indentation size effect (ISE) can result from instrumental conditions or modification of the near-to-surface layers during surface preparation of the sample under study. For example, contamination or work-hardening of such layers during polishing/grinding, inappropriate surface roughness, incorrect indent area-function calibration, and indenter tip blunting are among the mechanisms yielding an increase in elastic modulus and hardness by nanoindentation at shallow depths.

**Figure 3:**
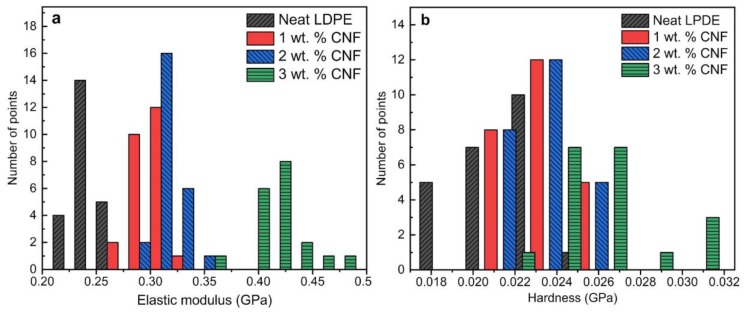
Histogram of 25 nanoindentation result points for (**a**) elastic modulus and (**b**) hardness values at 0.5 mN load resulting in about 1000 nm indentation depth.

Figure 3 illustrates a histogram of 25 data points collected from the nanoindentation tests for each sample tested at 0.5 mN load. At this load, the resulting penetration depth was around 1000 nm for neat LDPE. As seen in Figure 3a,b, the results are highly scattered which can be due to several reasons such as uneven surface roughness, non-uniform dispersion of CNFs and semicrystalline structure of the polymer composite. Although a very fine surface roughness of 15 nm was achieved, scratches made by removed CNFs and other polishing residuals still exist. Local surface peaks and valleys caused by these scratches could be another source of variation in NI results. Therefore, the indentation areas were selected manually to avoid surface scratches taking surface roughness out of possible reasons for high scattering of the NI data. The distribution of the data points indicates that each indent may possibly hit a high CNFs concentrated area or a non-reinforced area leading to a deviation in the material‘s behavior against penetration. This will cause a high scattering of the data points around a peak value. However, the peak values represent the general behavior of the composite. Interestingly, it can be observed that the range in which all data points for a specific sample is present is improving with the addition of CNFs to the polymer matrix. This clearly indicates the contribution of CNFs in improving the elastic modulus of the LDPE (Figure 3a). Hardness values for all four samples were very concentrated in one area, showing that the addition of CNF did not significantly improve the hardness (Figure 3b). Although there were few points where 3 wt. % CNF showed high hardness values, as a whole, the peak values for three samples were located very close at one area.

**Figure 4:**
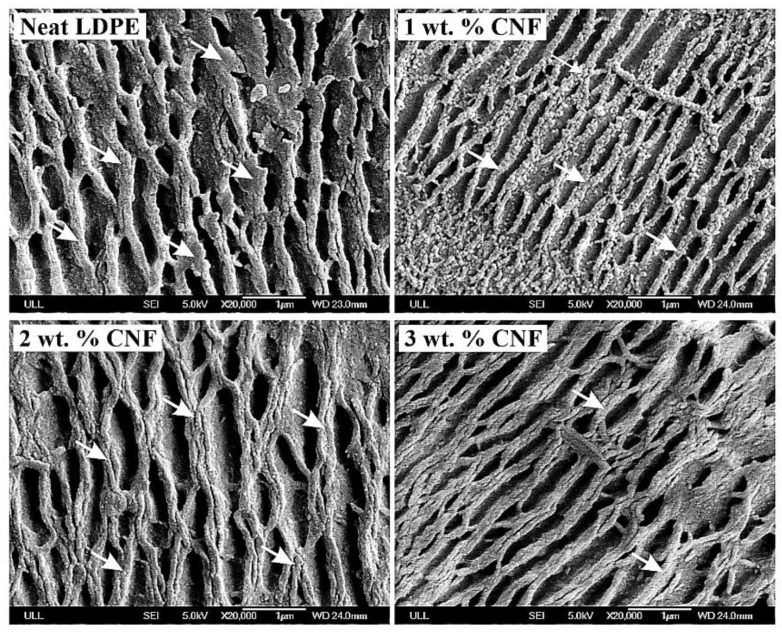
SEM micrographs of the samples after illustrating the semicrystalline morphology of LDPE with high branching features; the dark regions show the amorphous regions which were removed by etching, and branches represent the crystal lamellas. It well discloses the very random probability of hitting amorphous regions versus crystalline regions while indenting which results in scattering of the nanoindentation data.

According to Klapperich et. al. [45], variations in the NI data could be due to the differences in the microstructure of the LDPE. Semicrystalline LDPE consists of crystalline regions surrounded by amorphous regions. Indentations can hit amorphous rich areas or crystalline areas which consequently result in variations in nanoindentation data. Microscopic analysis was performed to better understand this concept where SEM micrographs of the surface of the samples after etching with potassium permanganate solution are given in Figure 4. Etching removed the amorphous region of the surface and exposed the semicrystalline structure of the LDPE nanocomposite. The dark areas in Figure 4 illustrate the amorphous parts which were removed by the etching process, and the light-colored branch-like structures (as indicated by arrows) demonstrate the crystalline morphology. This disorganized lamella structure of low-density polyethylene was previously reported by researchers for melt crystalized specimens [46].

**Figure 5:**
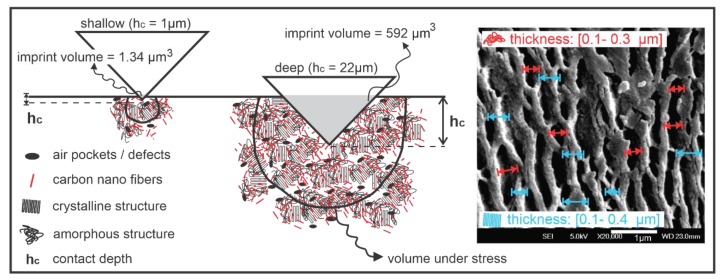
Schematic illustration of the indentation area for low and high penetration depth: Higher indentation depth results in bigger volume under the test which includes more characteristic features of the nanocomposite such as nanofillers, nano/micro defects and degree of crystallinity.

Since this study aims to obtain reliable NI data and compare them with the data obtained through a conventional tensile test, a high indentation depth was chosen for the load-control method to achieve reproducible mechanical properties. No thickness restriction for the samples in this study, contrary to the case of thin films, made it possible to have no limits on the penetration depth in a range of microns. By increasing the penetration depth, the volume under indentation increases. Volume under the test (VUT) includes part of the bulk material which is under stress caused by the indenter tip and reacts against penetration. A bigger VUT is a better representation of the bulk material in regard to the inclusion of the nano/micro defects existing in the structure of the nanocomposite. These defects could include air pockets, CNF agglomerations, microcracks, and any other defects created during injection molding. Figure 5 schematically shows the effect of a larger VUT for a better representation of the sample properties.

The residual volume of the indent in the NI test can roughly give an idea of the VUT. Although the volume of the residual indent is much smaller that the VUT, it can be used to compare the sizes of VUTs at different depths. Volume of the prints can be calculated indirectly from the load-depth curve and the indenter geometry using [47]:

(4) $$V=\frac{1}{3}\left(A_{p}\times h_{c}\right)$$

where $A_{p}$ is the plastic contact area, and $h_{c}$ is the final depth of the residual impression after unloading. Although it is not an exact method of calculation of the volume of the imprints since it does not take into account the elastic recovery of the surface, pile-up, or sink-in, it is still accurate enough to compare the imprint volume sizes at different loads. The residual volume for loads of 0.5 and 200 mN for LDPE are calculated as 1.34 and 592 μm^3^, respectively. For comparison purposes, the volume of a single carbon nanofiber used in this study with 100 micrometer length is about 7 μm^3^. It can be concluded that, for a semicrystalline structure of CNF/LDPE, the very small VUT at 0.5 mN cannot be a good representation of the bulk structure of the nanocomposite. This is believed to be a source of variations in NI data measured at very low depths in ranges of hundreds of nanometers for all semicrystalline polymer nanocomposites.

**Figure 6:**
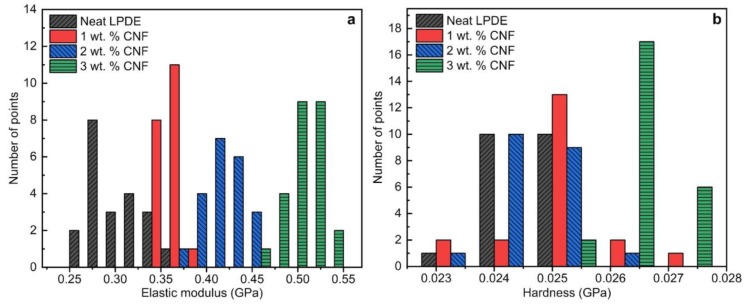
Histogram of 25 nanoindentation result points for (**a**) elastic modulus and (**b**) hardness values at 200 mN load resulting in 20 µm indentation depth.

Considering all the above discussions, indents were repeated at a significantly higher load (200 mN), which resulted in indentation depths of 22 microns (Figure 6). Table 3 compares mean, standard deviation and coefficient of variations (COVs) of the 25 data points for nanoindentation results at two loads of 0.5 and 200 mN. As expected, the distribution of the data points for deep indents reveals the consistency of the results and a significant improvement in COV of the data points in comparison to the shallow indents. Our data suggest that wherever the restrictions of thickness of the samples allows, it is most favorable to conduct NI tests deeper to obtain more consistent and repeatable results.

**Figure 7:**
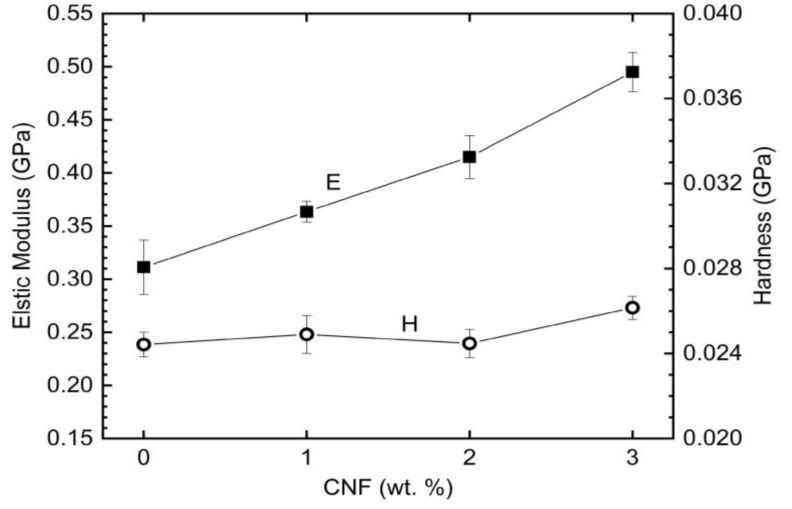
Elastic modulus and hardness obtained at 200 mN penetration load.

CNF has a very high aspect ratio (around 1000) which enhances the mechanical strength of the LDPE matrix by transferring applied load. We previously reported that carbon-based nanoparticles such as CNTs and CNFs can act as a nucleating agent and enhance the crystallization of the LDPE which indicates a superior adhesion of LDPE to carbon nanoparticles [12]. A good fiber-matrix interface plays an essential role in load transfer, resulting in mechanical strengthening of the polymer matrix. Figure 7 represents the elastic modulus and hardness for neat LDPE and CNF/LDPE nanohybrids obtained by nanoindentation at 200 mN load. Our results revealed that the addition of CNFs to LDPE contributes to a significant increase in the elastic modulus where the addition of 3 wt. % CNF to LDPE improves elastic modulus by 59%. However, hardness (*H*) of LDPE increases only by 12% by adding 3 wt. % CNF. The lower *H* increment compared to *E* has been reported by several researchers [48]. Diez-Pascual et al. [1] reviewed *E* and *H* evolution of different polymer nanocomposites and concluded that carbon nanofillers are more effective in enhancing the stiffness than the hardness of thermoplastic matrices, and H increments are primarily lower than those of E. In general, the results suggest that for thermoplastic matrices, interfaces and the degree of dispersion of CNFs/CNTs have different effects on the elastic and plastic response of such nanocomposite systems but the reasons for such behavior are not clear for the writer and need further investigations (to read more refer to page 45 of Ref. [1]).

**Figure 8:**
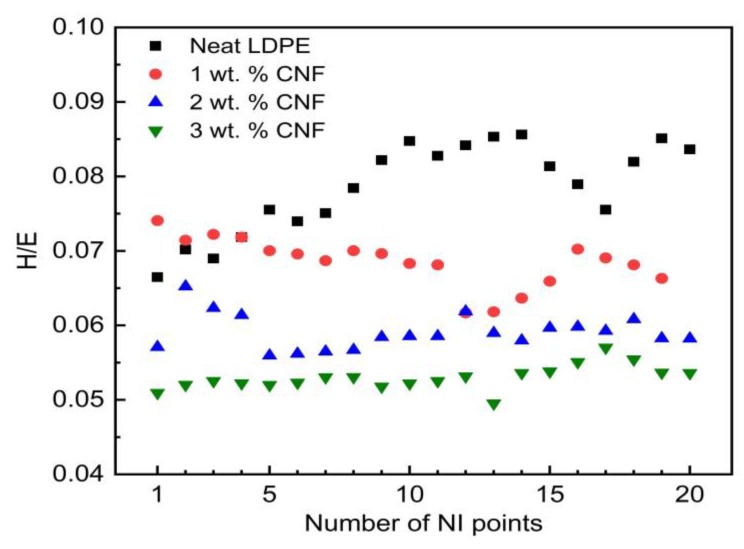
Hardness-to-modulus ratio as an index of resistance to wear.

Figure 8 presents the *H/E* ratios derived from the nanoindentation tests for CNF/LDPE samples. The ratio between *H* and *E* has been found to be a great tool to describe performance criteria in wear resistance of a material, particularly for coated materials [49]. Elastic strain to failure, the critical yield pressure for plastic deformation, and the fracture toughness can be determined by *H/E* values. As given in Figure 8, the *H/E* ratios for the neat LDPE samples are the highest and for the 3 wt. % CNF samples are the lowest. This reveals that CNFs are not great additions to LDPE where wear resistance is important. This is because, as shown earlier, the *E* increments are comparatively bigger than *H* increments, contributing lower *H/E* values.

### 3.3. Local Mapping of Mechanical Properties

**Figure 9:**
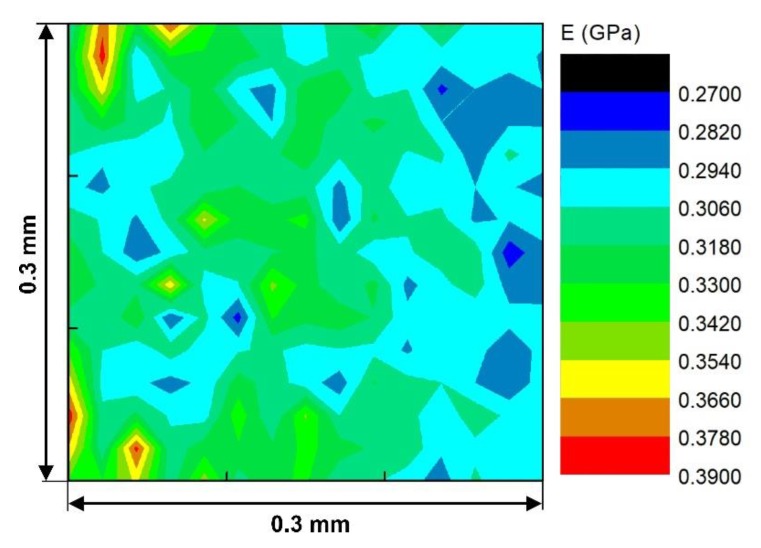
Map of 225 points of elastic modulus of the 2 wt. % CNF/LDPE sample in a 0.3 mm × 0.3 mm area at 0.5 mN load.

Figure 9 exhibits a 2-D contour plot of elastic modulus for a randomly-chosen surface area. It shows a map of 225 points of elastic modulus for arbitrarily chosen 2 wt. % CNF sample on an area of 0.3 mm × 0.3 mm. Indents were made on a grid of 15 × 15 points. The distance between each two adjacent points was 15 µm to ensure that there is enough space to eliminate the effects of one indent on its neighboring points. Red zones in the map (as indicated by arrows) show zones with high E values which could be a result of higher concentration of CNFs (with assuming equal surface roughness throughout the surface and CNFs as the dominant factor in changing the E) and dark blue zones (indicated by circles) with less concentration of CNFs. This function of nanoindentation technique in monitoring the distribution of mechanical properties in a specific area could have potential in biomedical applications where mechanical properties are required to be monitored locally [50]. 

For instance, in a knee prosthesis, which is designed to experience varying local stresses, it is of the utmost importance to know that the corresponding areas have enough strength to withstand the loads and a localized mapping of the mechanical properties such as *E* and *H* values could be beneficial.

### 3.4. Comparative Analysis between Nanoindentation, Tensile Test and Halpin-Tsai Results

It is extremely difficult to compare the nanoindentation results with those achieved by conventional tensile test results. Apart from the sources of error in depth-sensing indentation (DSI) experiments, there are big differences between nanoindentation and tensile test results: (1) there is a huge difference between the volumes under the tests; a conventional tensile test sample might include significant amounts of micro/macro size defects such as air pockets, micro cracks, and structural defects, which are less probable to exist in the relatively small volume under the test in the DSI experiments. (2) The stress and strain conditions in the two techniques are quite different; the uniaxial load direction in tensile test and the radially evolving load direction in DSI tests induce different stress and strain states which totally differ from the principles of measurements methods for the two techniques. So, the discrepancies between the results from the DSI and macro techniques are expected. In addition, for reinforced nanocomposites, this disparity of the results is expected to be larger because the longer nanofillers tend to orient along the load direction in tensile tests, resulting in higher reinforcement comparing to the DSI tests [1].

**Figure 10:**
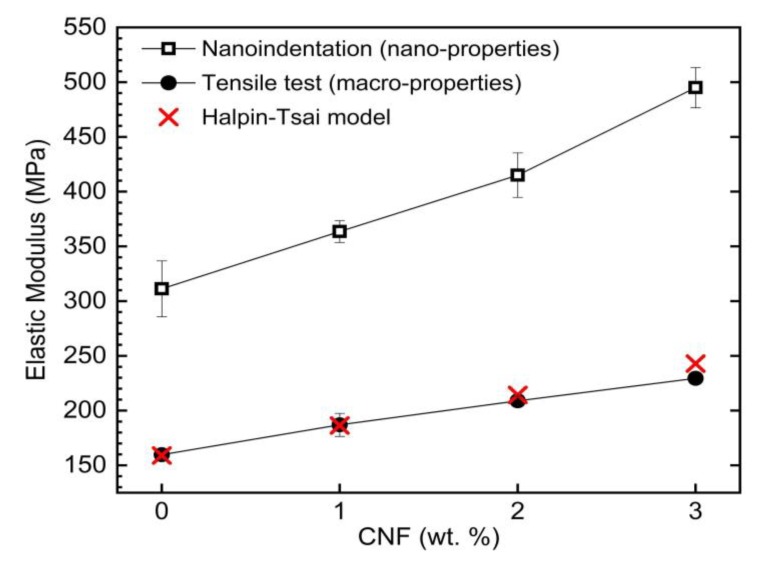
Comparison of experimental and theoretical values of elastic modulus for CNF/LDPE samples obtained by nanoindentation, tensile test results by Khattab et al. [14] and Halpin-Tsai model.

Figure 10 shows the elastic modulus obtained by nanoindentation tests compared with the tensile test results from our previous work [14] as well as the theoretically calculated results using the Halpin-Tsai model. It can be observed that elastic modulus obtained by nanoindentation tests are almost more than double than that obtained by the conventional tensile test. This phenomenon has been reported for polymeric materials repeatedly in the literatures. For instance, Tranchida et al. [24] reported a 70% increase in modulus for polystyrene (PS) and 64% increase for polycarbonate. Lu et al. [51] reported a 67% increase for poly (methyl methacrylate), while VanLandingham et al. [52] reported an increase of 20% for poly (benzocyclobutene) in comparison to macro properties. The authors also reported that the trend of changing E by the addition of carbon nanofillers is almost the same for both techniques. This can be clearly seen in Figure 10 that the trend of change in E values with the addition of CNF remained the same for the two techniques. The addition of 1, 2 and 3 wt. % CNF to LDPE resulted in 17%, 33% and 59% increase in E measured by nanoindentation and 17%, 31% and 44% increase in E measured by conventional tensile test, respectively. 

The Halpin-Tsai model, which is based on a self-consistent micromechanics method, was utilized to calculate *E* of CNF/LDPE systems with different nanofiller concentrations. The equation below is derived for a randomly-oriented short fiber-reinforced polymer composite [53]:

(5) $$E_{c}=E_{p}\left[\frac{3}{8}\left(\frac{1+\left(\frac{2a}{3}\right)\eta_{L}V_{CNF}}{1-\eta_{L}V_{CNF}}\right)+\frac{5}{8}\left(\frac{1+2\eta_{T}V_{CNF}}{1-\eta_{T}V_{CNF}}\right)\right]$$

(6) $$\eta_{L}=\frac{\left(E_{CNF}/E_{p}\right)-1}{\left(\frac{E_{G}}{E_{p}}\right)+\frac{2a}{3}}$$

(7) $$\eta_{T}=\frac{\left(E_{CNF}/E_{p}\right)-1}{\left(\frac{E_{CNF}}{E_{p}}\right)+2}$$

where, $E_{c}$ and $E_{p}$ are the elastic modulus of the nanocomposite and polymer (LDPE) respectively. The parameter a in Equations (5) and (6) is the aspect ratio of CNF equal to $l_{CNF}/t_{CNF}$, with $l_{CNF}$ as the average length of the fibers and $t_{CNF}$ as their average diameter. This resulted in an a value of 166 for CNFs in this study. $V_{CNF}$ is the volume fraction of the CNFs which needs to be calculated from the weight percentage (wt. %) with the following equation:

(8) $$V_{CNF}=\frac{w\;\rho_{p}}{w\;\rho_{p}+\left(1-w\right)\rho_{CNF}}$$

where, $\rho_{p}$ and $\rho_{CNF}$ represent the density of LDPE and CNF, taken as 0.9 and 2.1 g/cm^3^, respectively. Kanagaraj et al. [54] used this model to evaluate E values of CNT-HDPE and found a close agreement of 2% deviation with the experimental results. Figure 10 indicates similarities between *E* values calculated by the HT model and macro-properties where relative deviation is under 2% for 1 and 2 wt. % CNF samples and 5% for 3 wt. % CNF samples. This indicates that the Halpin-Tsai model can be a good prediction tool to estimate the macro properties of CNF/LDPE nanocomposites with high accuracy.

## 4. Conclusions

The load-control method was used to study the surface mechanical properties of CNF/LDPE nanocomposite systems. The designed stepwise grinding/polishing procedure, which consisted of a combination of SiC and microcloth paper utilization with a specified rotating speed, time and lubricant (details are given in Table 2), was shown effective to obtain very fine surface finishes ($R_{m}$ = 15 nm) for the soft CNF/LDPE systems. Nanoindentation data obtained at two different loads of 5 and 200 mN showed that the scattering of data points was dependent on the penetration depth. It was shown that deeper penetration results in higher volume under stress/strain and consequently has higher probability to include the characteristics of the bulk material structure such as micro/macro defects, air pockets, CNF agglomerations, and degree of crystallinity. It was observed that the COV of the NI data were decreased from 7.2% to 3.7%, while the penetration depth was increased from 1 to 22 µm. Moreover, microscopic analysis using SEM micrographs and NI imprint volume calculations revealed that NI at the submicron scale (tens or hundreds of nm) may result in high variations in measuring mechanical properties of semicrystalline CNF/DLPE systems. Therefore, this study suggests for semicrystalline polymer nanocomposites to be tested at micrometer scale (indentation depths of a few microns) where allowed to achieve more repeatable and statistically-reliable data.

*E* calculated by NI experiments for CNF/LDPE systems were found to be twice as big as the *E* obtained by the conventional tensile test data which were found to be in close agreement with the Halpin-Tsai model. This deviation, which was reported for several polymeric materials in preceding studies [33,34,35], was deemed to have several disparities: (1) different stress/strain states and principles of calculation; (2) significant difference between the volumes under the test; (3) and consequently, large difference in inclusion of micro/macro scale defects and CNF agglomerations. E and H obtained by nanoindentation revealed that CNFs were more effective in enhancing the stiffness of LDPE than the hardness. *H/E* ratio of LDPE decreased by the addition of CNF, revealing that CNF is not a good addition to LDPE systems where wear resistance is of great importance.

## Figures and Tables

**Table 1:** Quantitative surface roughness results from AFM for methods A, B, and C.

	Method A	Method B	Method C
Image Z range	608.4 nm	464.2 nm	125.7 nm
Image Rms	65.7 nm	38.7 nm	18.72 nm
Image mean roughness ($R_{m}$)	50.0 nm	28.0 nm	15.0 nm

**Table 2:** Summary of the steps adopted for methods A, B and C for polishing of the CNF/LDPE samples.

Paper	Speed (rpm)	Time (minutes)	Lubricant
**Method A**			
SiC–P200	180	5	Water
SiC–P400	180	8	Water
SiC–P800	180	8	Water
SiC–P1200	180	10	Water
SiC–P2500	180	10	Water
**Method B**			
SiC–P400	180	5	Water
SiC–P600	180	8	Water
SiC–P1200	180	8	Water
SiC–P2500	180	8	Water
Microcloth	160	8	Diamond suspension 1 μm size
Microcloth	160	8	Alumina suspension 0.05 μm size
**Method C**			
SiC–P400	150	5	Water
SiC–P800	150	8	Water
SiC–P1200	150	8	Water
SiC–P2500	180	8	Water
Microcloth	180	12	Diamond suspension 1 μm size
Microcloth	180	12	Alumina suspension 0.3 μm size
Microcloth	180	12	Alumina suspension 0.05 μm size

**Table 3:** Elastic modulus (*E*) and hardness (*H*) for CNF/LDPE samples at 0.5 and 200 mN load.

CNF (wt. %)	$0.5\;\mathrm{mN}\;(h_{c}\;=\;1\;\mu m)$						$200\;\mathrm{mN}\;(h_{c}\;=\;22\;\mu m)$					
	*E*			*H*			*E*			*H*		
	Av.	Std.	cov%	Av.	Std.	cov%	Av.	Std.	cov%	Av.	Std.	cov%
**0**	0.25	0.01	5.17	0.022	0.002	8.001	0.31	0.03	8.22	0.024	0.001	2.387
**1**	0.30	0.02	5.04	0.023	0.001	4.809	0.36	0.01	2.73	0.025	0.001	3.570
**2**	0.32	0.03	8.49	0.023	0.001	5.872	0.42	0.02	4.90	0.024	0.001	2.710
**3**	0.42	0.05	10.76	0.025	0.002	9.319	0.50	0.02	3.72	0.026	0.001	2.074

## Supplementary Materials

The following are available online at https://www.mdpi.com/2079-4991/9/10/1357/s1, Figure S1: Representative load-displacement curve with no “nose” effect with holding time as 50 seconds, Figure S2: SEM image of a single indent in 2 wt. % CNF/LDPE showing no pile-up effect.
