# Supplementary material for: Surface Mechanical Characterization of Carbon Nanofiber Reinforced Low-Density Polyethylene by Nanoindentation and Comparison with Bulk Properties
Source: Nanomaterials (Basel). 2019 Sep 22;9(10):1357. doi: 10.3390/nano9101357 (PMC6835558; doi:10.3390/nano9101357)
Supplement: Supplementary file 1 [file nanomaterials-09-01357-s001.pdf]

Supplementary material:

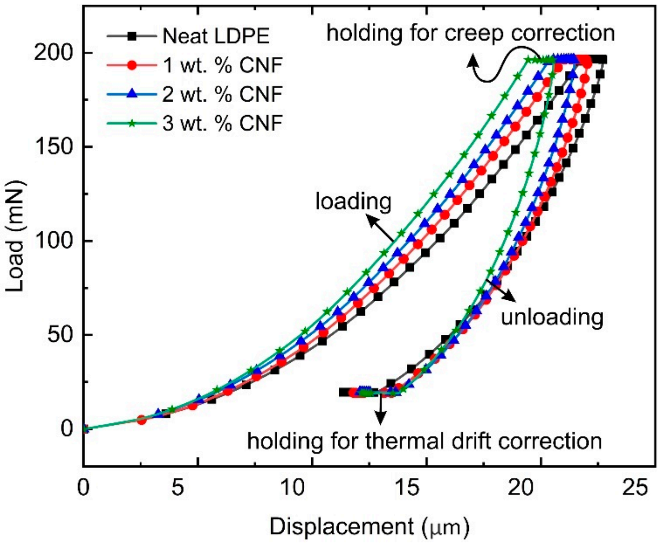


Figure S1. Representative load-displacement curve with no "nose" effect with holding time as 50 s.

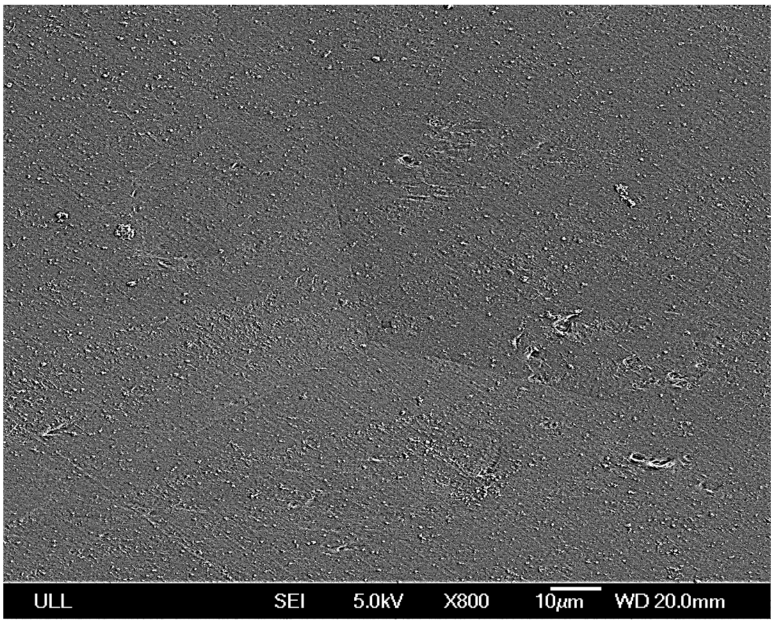


Figure S2. SEM image of a single indent in 2 wt. % CNF/LDPE showing no pile-up effect
